# Empirical research of foreign direct investments efficiency in the European Union on the edge of pandemic outbreak

**DOI:** 10.1371/journal.pone.0313161

**Published:** 2025-01-08

**Authors:** Milan Džogan, Roman Lacko, Zuzana Hajduová

**Affiliations:** 1 School of Economy, Shanghai University, Shanghai, P.R. China; 2 Department of Tourism, Faculty of Commerce, University of Economics in Bratislava, Bratislava, Slovakia; 3 Department of Business Finance, Faculty of Business Management, University of Economics in Bratislava, Bratislava, Slovakia; University of Almeria: Universidad de Almeria, SPAIN

## Abstract

The study focuses on the effects of the COVID-19 pandemic on the economy and foreign direct investments in the European Union countries. Using data envelopment analysis constant and variable returns to scale models, and the Malmquist productivity index, we measured the efficiency of economic resource utilization concerning foreign direct investments and gross domestic product. These efficiencies were assessed in 2019, before the full outbreak of COVID-19, and in 2020, when uncertainty and the consequences of the pandemic were most pronounced. Despite the challenges posed by the pandemic, the European Union displayed economic resilience in the first year, with no significant reduction in foreign direct investment efficiency. Lower-income European Union countries saw an increase in foreign direct investment efficiency, attributing this to positive changes in managerial efficiency. This suggests that decisions made by authorities positively impacted foreign direct investment flows in economically less developed European Union regions despite restrictive measures and market uncertainties.

## Introduction

The global pandemic of COVID-19 has had a profound impact on many areas of life, including the economy. In a relatively globalized world, the disease spread within a few months and caught the world unprepared. The once-globalized world with open borders suddenly turned into a world of closed countries and regions. This led to not only a health crisis but also an economic one [1]. During crises, there is a decline in foreign direct investments (FDI) [2], yet what is desirable is precisely an increase in investments [3].

Significant uncertainty prevailed in the pandemic’s early stages [4]. The relationship between uncertainty and FDI is not a widely discussed scientific topic, which may also be due to a lack of comparable indicators. However, the authors demonstrate that domestic uncertainty does not have a significant impact on FDI, unlike global uncertainty [5], which was the case during the COVID-19 pandemic. Countries were affected by global uncertainty, which translated into local uncertainty in countries around the world. At the onset of the COVID-19 pandemic, many countries implemented expansive monetary policies to support the economies severely affected by the pandemic’s consequences. This primarily involved reducing interest rates, positively impacting FDI flows [6]. On the other hand, various restrictions, uncertainties, and issues in supply chains and transportation of goods (due to border closures) resulted in a decline in FDI in many countries worldwide [1, 7, 8]. Less developed countries struggled with reducing FDI inflows [8–10]. This decline mainly affected the service sector, predominantly in emerging countries dependent on FDI inflows [11]. Moreover, this effect was more severe for greenfield investments and significantly weaker for cross-border mergers and acquisitions [12]. Even studies indicate a significant decrease in FDI inflows in countries with more infections [13].

In recent years, numerous studies have focused on the impact of the COVID-19 pandemic globally [14, 15]. The effects of the pandemic varied among countries worldwide, stemming from economic heterogeneity and different approaches to addressing the pandemic [16]. However, some research confirms that the consequences of the pandemic for major world economies in terms of FDI may not have been as devastating as initially anticipated during the early stages of the pandemic [17]. Still, strong economies had to adopt specific measures to redirect FDI exports to regions less affected by the pandemic [15]. FDI has also proven to be a path for the economic development of countries after a severe crisis associated with the pandemic [18].

The inflow and outflow of FDI are crucial for individual country economies and political entities [19], even though they come with several adverse effects, such as increasing CO_2_ emissions [20]. Numerous studies have explored the relationship between FDI and the European Union (EU) [21–24]. However, very few studies have comprehensively addressed the issues and changes in FDI during the COVID-19 pandemic. Predominantly, we encounter research that measured the effects of the pandemic in other regions of the world [25, 26] or research that did not focus on the pandemic years [27]. Some studies addressed certain EU countries as emerging countries [28]. The EU is one of the most significant political entities in which FDI flows and serves as a source market for FDI. It is also a region significantly affected by the pandemic. For example, FDI in the tourism sector in the EU (Spain, Germany, Italy, and other countries) is more significant [29]. This sector was one of the most affected overall. The uncertainty arising from the pandemic impacts demand and the openness of international trade [25, 30]. Moreover, the EU is a political entity whose members are still a relatively heterogeneous group, although its main objective is the cohesion of the member countries [22, 31]. Therefore, we believe that examining the impacts of the pandemic and uncertainty on FDI is crucial for understanding the processes related to FDI in the EU as a whole and in individual countries or groups of countries. A thorough understanding of the causes and consequences of economic disruptions can help formulate policies that enhance the resilience of foreign trade against future crises. Policies of countries worldwide should focus on preventing future crises, not only of a pandemic nature [14].

Hence, this study focuses on assessing the effects of the COVID-19 pandemic on the efficiency of FDI using the EU countries as a case study object. This study f to determine the impact that the COVID-19 pandemic had on the efficiency of EU countries in terms of both FDI inflow and outflow at the onset of the pandemic. Based on these findings, the study aims to evaluate differences among countries that are part of a single market but still exhibit distinct economic conditions.

## Materials and methods

### Methods and variables

Measurement of the efficiency of fundamental economic factors on outputs in terms of both inward and outward FDI requires specific methodological approaches. One of the most commonly used methods for measuring technical efficiency at the international level is the Data Envelopment Analysis (DEA) method [32]. This method has also proven effective in measuring selected effects in the field of FDI [33–35].

For this study, we decided to use the input-oriented constant returns to scale (CRS) model (1), which is expressed as follows:

$$\begin{array}{c} \underset{\theta_{B},\lambda }{\text{min}}\theta_{B}\\ \text{s}.\text{t}.\theta_{B}{\text{x}}_{o}-\text{X}\lambda \geq 0\\ \text{Y}\lambda \geq {\text{y}}_{o}\\ \lambda \geq 0. \end{array}$$ (1)

For completeness and measurement under variable returns to scale (VRS) conditions, we will also use the input VRS model, the form of which is as follows (2):

$$\begin{array}{c} \underset{\theta_{B},\lambda }{\text{min}}\theta_{B}\\ \text{s}.\text{t}.\theta_{B}{\text{x}}_{o}-\text{X}\lambda \geq 0\\ \text{Y}\lambda \geq {\text{y}}_{o}\\ e\lambda =1\\ \lambda \geq 0, \end{array}$$ (2)

Where (for models 1 and 2) θ_*B*_ is the efficiency score being evaluated, x_*o*_ is the vector of inputs, y_*o*_ is the vector of outputs, λ is the weights assigned to evaluated decision-making units (DMU), X is the matrix of inputs, Y is the matrix of outputs and e is the vector of ones. The above-mentioned models will help us obtain values of input technical efficiencies, whose values belong to a closed interval between 0 and 1. For a more detailed description of the models, refer to [32, 36].

To measure changes over time, we will also use the Malmquist Production Index, which can be mathematically expressed by the following formula (3):

$$MPI_{I}^{G}={\left(ME_{I}.TE_{I}^{G}\right)}^{1/2}=\left(\frac{E_{I}^{t+1}\left(x^{t+1},y^{t+1}\right)}{E_{I}^{t}\left(x^{t},y^{t}\right)}\right).{\left[\left(\frac{E_{I}^{t}\left(x^{t},y^{t}\right)}{E_{I}^{t+1}\left(x^{t},y^{t}\right)}\right).\;\left(\frac{E_{I}^{t}\left(x^{t+1},y^{t+1}\right)}{E_{I}^{t+1}\left(x^{t+1},y^{t+1}\right)}\right)\right]}^{1/2}$$ (3)

Where ${MPI}_{I}^{G}$ refers to geometric mean of Malmquist productivity index, *ME* is managerial change in efficiency, and *TE* is change in technological efficiency, *E* is efficiency, *I* is the number of the DMU, *t* is time period to be evaluated, *x* is the set of inputs and *y* is the set of outputs, for deeper description see [37]. The division of MPI into two aspects is crucial for understanding determinants of overall efficiency change in selected periods. Technical Efficiency measures whether a DMU is operating efficiently by comparing it to the best possible performance (the production frontier). If a DMU is not on this frontier, it means there are inefficiencies in converting inputs into outputs. Identifying these inefficiencies can help enhance operational processes and better use of resources. Managerial efficiency assesses whether the DMU uses the right combination of inputs to produce its outputs. It looks at whether the proportions of inputs are optimal for achieving the desired outputs. By adjusting the input mix, a DMU can potentially improve its overall performance [38].

One of the most crucial processes in measuring efficiency is the selection of variables. In this study, we will assume that the proper combination of inputs—labor, capital, and land—can improve FDI inward and outward flows. This novel approach can contribute to a complex view of FDI flows and the comparability of countries in different situations of FDI openness.

After thoroughly examining previous studies, we decided to use the following indicators, based on the trends and commonly used variables as inputs and outputs for our model [39, 40], as presented in Table 1.

**Table 1:** Description of variables.

	Variable name	Variable symbol	Units	Source
Input	Gross fixed capital formation	GFCF	bn. EUR	Eurostat
	Energy consumption in the industry	ECi	tons of oil equivalent	Eurostat
	Energy consumption in services	ECs	tons of oil equivalent	Eurostat
	Human capital	HC	thousands	Eurostat
Output	FDI inward stocks	FDIi	bn. USD	UNCTAD
	FDI outward stocks	FDIo	bn. USD	UNCTAD
	Gross domestic product	GDP	bn. USD	Eurostat

Source: Own processing.

For input variables in our DEA model measuring FDI efficiency, we determine the use of essential factors of production such as capital, energy, and labor. Therefore, the first input representing capital is Gross fixed capital formation (GFCF), commonly used in the DEA analysis as a capital proxy [14, 41–43]. For the energy variable, which denotes the cost and availability of substitute energy, we choose to divide energy consumption in industry (ECi) and energy consumption in services (ECs) [40]. Moreover, if a country can produce more GDP using less energy inputs, it could measure the energy efficiency effects of FDI. The last input variable is human capital (HC), which represents the number of people employed in a country and hints at the workforce capacity [44, 45].

As output variables, we decided to use inward foreign direct investment (FDIi) and outward foreign direct investment (FDIo) and Gross domestic product (GDP), as FDI alone may not reliably describe the level of the problem under research. These variables are commonly used to measure countries’ efficiency in terms of FDI [40, 46, 47].

### Data and research object

This research is based on evaluating the FDI efficiency of 24 EU countries in 2019 and 2020 through the DEA. In the initial research stage, the idea was to assess every country in EU27. However, after several errors and complications, Malta, Cyprus, and Luxembourg were excluded from the total current EU27 members, as their values are not homogenous with other, larger member countries, and the DEA method encountered anomalies in the final efficiency categorization. This phenomenon is not exceptional in our study and has already been recorded in several previous studies comparing the efficiency of EU27 countries. Data used in this study were obtained from publicly available Eurostat and UNCTAD databases [48, 49]. We then created a panel of 48 DMUs, which will be measured in the case of DEA analysis by the DEA Window approach [50]. All the data are available in the Supporting information section—S1 Table. Input and output variables.

## Results

In the first step of the research, we computed the efficiency values of the selected EU countries. Table 2 presents DEA CRS and VRS values for 2019 and 2020. Countries are sorted according to their efficiency values.

**Table 2:** Results of DEA CRS and VRS model.

Country	CRS			Country	VRS		
	2019	2020	Diff		2019	2020	Diff
Netherlands	0.9301	1.0000	0.0699	Poland	0.8387	0.9236	0.0849
Slovakia	0.6639	0.7222	0.0583	Slovakia	0.6979	0.7688	0.0709
Poland	0.6478	0.7036	0.0558	Lithuania	0.8789	0.9341	0.0552
Lithuania	0.6677	0.7155	0.0478	Ireland	0.9669	1.0000	0.0331
Slovenia	0.8380	0.8796	0.0416	Slovenia	0.9827	1.0000	0.0173
Ireland	0.9618	1.0000	0.0382	Czechia	0.5885	0.6004	0.0119
Czechia	0.5860	0.5991	0.0131	Bulgaria	0.7513	0.7627	0.0114
Romania	0.8153	0.8157	0.0004	Romania	0.8183	0.8207	0.0024
Bulgaria	0.6729	0.6731	0.0002	Denmark	1.0000	1.0000	0.0000
Denmark	1.0000	1.0000	0.0000	Estonia	1.0000	1.0000	0.0000
Sweden	0.8547	0.8524	-0.0023	France	1.0000	1.0000	0.0000
Portugal	0.7887	0.7858	-0.0029	Germany	1.0000	1.0000	0.0000
Finland	0.8644	0.8604	-0.0040	Netherlands	1.0000	1.0000	0.0000
Spain	0.8923	0.8830	-0.0093	Sweden	0.9032	0.9016	-0.0016
Italy	0.9018	0.8908	-0.0110	Finland	0.8685	0.8648	-0.0037
Hungary	0.5806	0.5695	-0.0111	Croatia	0.8085	0.8044	-0.0041
Germany	0.8844	0.8730	-0.0114	Portugal	0.7951	0.7859	-0.0092
Austria	0.8881	0.8762	-0.0119	Hungary	0.5906	0.5808	-0.0098
Belgium	0.8681	0.8550	-0.0131	Italy	1.0000	0.9898	-0.0102
France	0.8376	0.8235	-0.0141	Spain	0.9822	0.9669	-0.0153
Latvia	0.5680	0.5446	-0.0234	Latvia	0.8566	0.8397	-0.0169
Croatia	0.6303	0.6061	-0.0242	Belgium	0.9181	0.8968	-0.0213
Estonia	0.6301	0.5961	-0.0340	Austria	0.9338	0.9100	-0.0238
Greece	1.0000	0.9533	-0.0467	Greece	1.0000	0.9642	-0.0358

Source: Own processing

Based on observations, it can be assessed that with the onset of the pandemic, most countries experienced a decline in efficiency values. The most significant decrease in CRS efficiency was recorded in countries such as Greece (-0.0467), Estonia (-0.0340), and Croatia (-0.0242). Despite challenging economic conditions, some countries were able to increase FDI efficiency levels during the pandemic. Interestingly, these are predominantly Eastern European countries, which belong to countries with lower efficiency values in our observed sample. The most significant increases in efficiency were observed in the Netherlands (+0.0699), Slovak Republic (+0.0583), and Poland (+0.0557). Other countries with increased efficiency include Lithuania, Slovenia, Ireland, Czech Republic, Romania, and Bulgaria. Denmark remained the only country with unchanged efficiency values, registering the highest efficiency in both years.

When evaluating the results of the DEA VRS method, it can be confirmed that the outcomes, when comparing increases or decreases in values, are very similar to the results of the CRS analysis. Greece recorded the most significant reduction in efficiency (-0.0358), while Poland (+0.0849), Slovak Republic (+0.0708), and Lithuania (+0.0552) experienced the highest increases in efficiency. Several countries, including Denmark, Estonia, France, Germany, and the Netherlands, achieved maximum efficiency using the VRS method.

**Fig 1:**
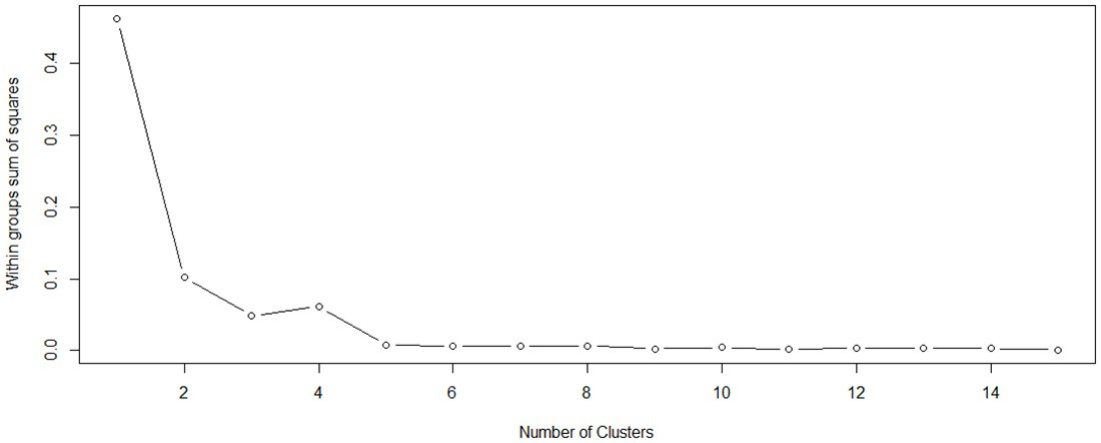
Results of clustering based on elbow method. Source: Own processing in R.

The elbow method analysis identified three main groups of countries based on similar attributes in the last year of the observed sample (see Fig 1). This method identifies the breaking point by visually assessing the created graph. It is one of the most commonly used methods for determining the number of clusters [51].

**Fig 2:**
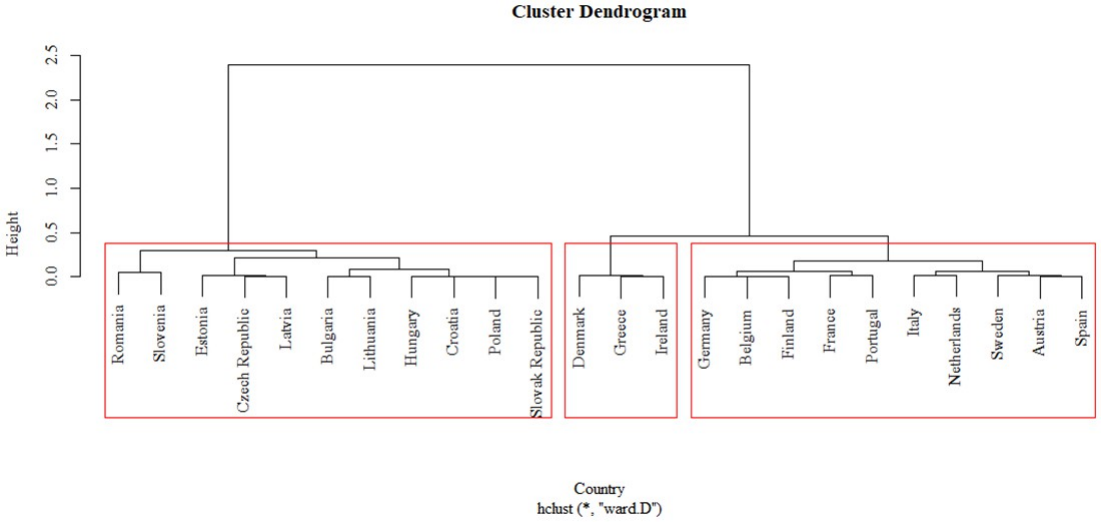
Dendrogram of CRS efficiency values. Source: Own processing in R.

**Fig 3:**
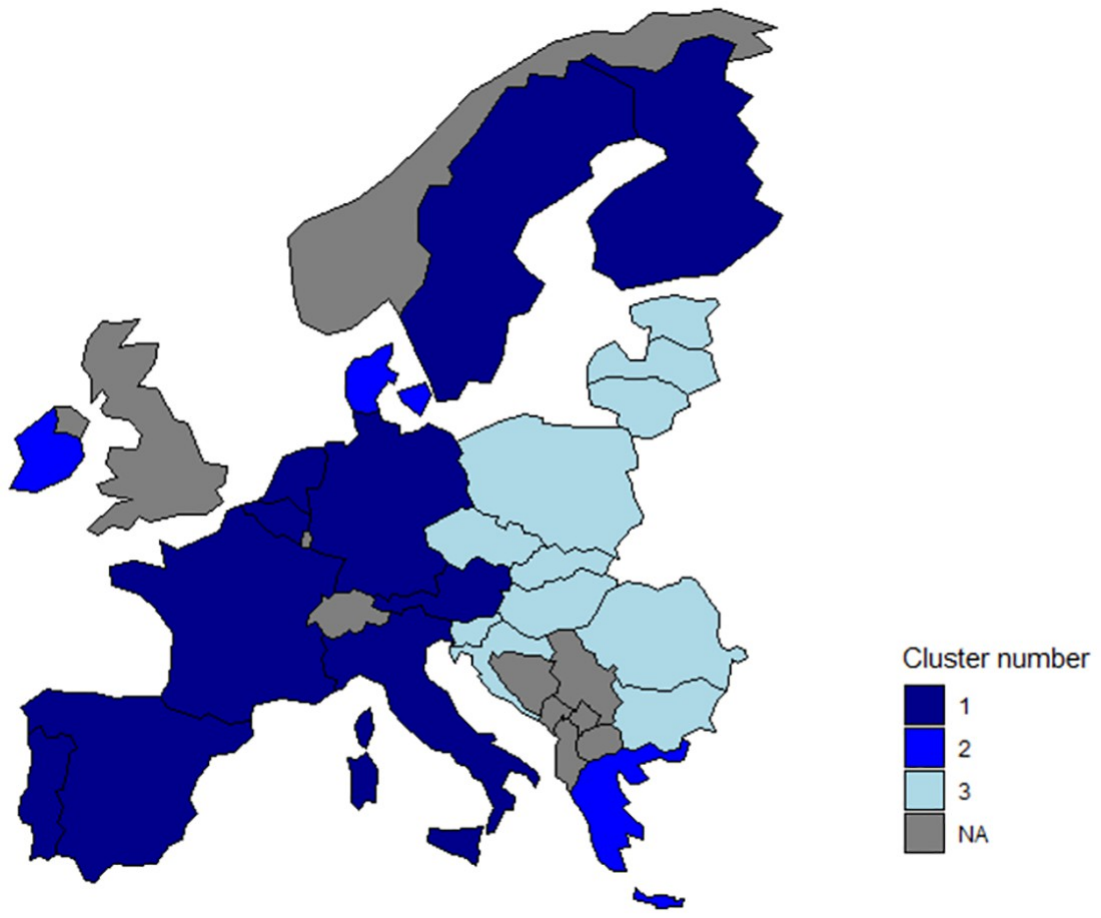
CRS efficiency clusters layout. Source: Own processing in MS Word.

Fig 2 presents the cluster analysis results for calculated FDI efficiency values, followed by a geographical layout in Fig 3 to understand differences across Europe better.

In the first cluster, we find countries in Eastern Europe that recorded the lowest FDI efficiency values and are historically and geographically close. This cluster includes countries with solid and long-standing political and economic ties, such as those in the Balkan region, the Visegrad Group (V4), and the Baltic countries. The second cluster is composed of countries Denmark, Greece, and Ireland. These countries achieved the highest efficiency values; geographically, they are predominantly island nations. The third cluster consists of Western and Northern European countries, characterized as nations with high economic, industrial, and living standards.

**Fig 4:**
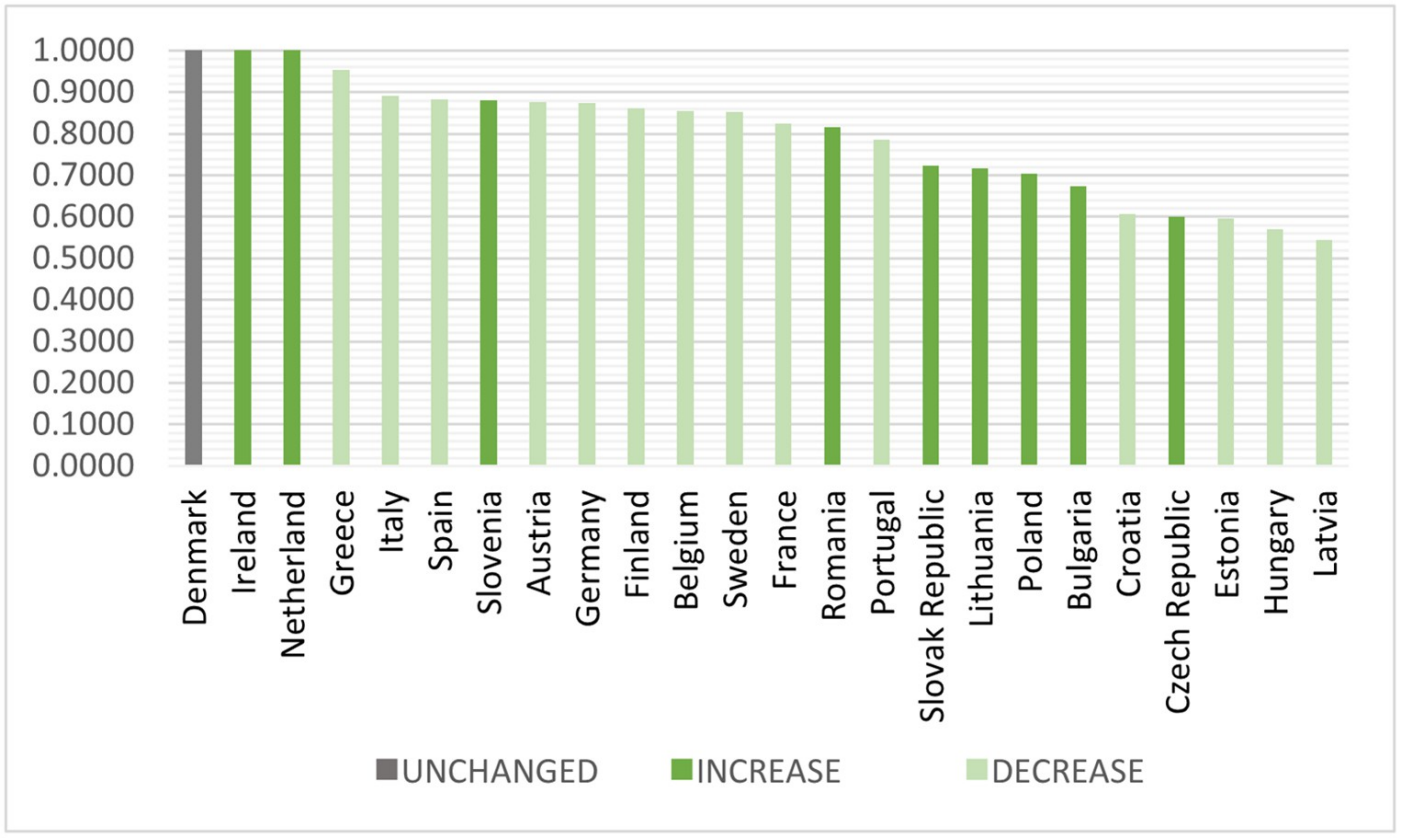
Total values of CRS efficiency 2020 downwardly. Source: Own processing.

To better visualize the differences in the observed years, we decided to present the results within categories where there was an increase, decrease, or, in one case, no change in efficiency. According to Fig 4, except for Ireland and the Netherlands, all seven remaining countries that experienced an increase in FDI efficiency come from the first cluster.

To examine changes in productivity, we utilized the Malmquist Productivity Index. Its decomposition helps us capture the reasons for changes in productivity over time, specifically focusing on managerial and technological factors influencing efficiency changes. The results are presented in Table 3.

**Table 3:** Results of Malmquist productivity index.

Country	2019–2020		
	MPI	ME	TE
Austria	0.9855	0.9572	1.0296
Belgium	0.9920	0.9778	1.0145
Bulgaria	1.0098	0.8990	1.1233
Croatia	0.9882	0.9915	0.9966
Czech Rep.	1.0245	1.0343	0.9906
Denmark	1.0040	1.0000	1.0040
Estonia	0.9809	0.9133	1.0741
Finland	0.9905	1.0225	0.9687
France	1.0018	1.0326	0.9702
Germany	0.9793	1.0153	0.9646
Greece	0.9436	1.0000	0.9436
Hungary	0.9900	0.9872	1.0028
Ireland	1.2422	1.0000	1.2422
Italy	0.9813	1.0083	0.9732
Latvia	0.9624	1.0157	0.9475
Lithuania	1.0644	1.0751	0.9900
Netherlands	1.4120	1.0000	1.4120
Poland	1.0475	1.0588	0.9893
Portugal	0.9933	0.9827	1.0108
Romania	0.9909	0.9989	0.9920
Slovak Rep.	1.0917	1.0900	1.0016
Slovenia	1.0501	1.0631	0.9878
Spain	0.9953	0.9919	1.0034
Sweden	1.0029	1.0142	0.9889

Source: Own processing

When evaluating the MPI results, we focus primarily on countries that experienced increased CRS efficiency values in 2020. In this case, we can confirm that Romania was the only one to register a slight decline in MPI (0.9909 / 0.91%). The highest MPI values were achieved by the Netherlands (1.4120), Ireland (1.2422), and the Slovak Republic (1.0917).

Furthermore, almost all Eastern European countries demonstrated a predominance in managerial efficiency, including the Czech Republic (1.0343), Lithuania (1.0751), Poland (1.0588), Romania (0.9989), the Slovak Republic (1.0900), and Slovenia (1.0631), with relatively less significant impact. Conversely, Bulgaria (1.1233), Ireland (1.2422), and the Netherlands (1.4120) showed a predominance in technological efficiency, with a much more pronounced impact.

## Discussion

Our results point to several interesting facts. This research has shown that a crisis can lead to streamlining FDI-related processes in less developed economies within the EU. From the perspective of this study, this could be caused either by 1) a reduction in inputs–production factors or 2) an increase in outputs, namely FDI flows and GDP. It is evident that in most countries, there was a decline in GDP in the first year of the pandemic. Of course, with a decline in GDP, there is also a decrease in production, leading to a decline in energy consumption. In some instances, there is also a decrease in human capital and capital formation. However, if there were a simultaneous decline in production factors, FDI flows, and GDP, efficiency in selected countries would certainly not have increased. Therefore, we conclude that behind the growth in efficiency is either an increase in FDI flows or at least maintaining their level, which is undoubtedly positive in times of crisis. This result contradicts research conducted in other regions of the world [8], highlighting the necessity of a different approach to EU policies.

However, this research has pointed out that more developed countries, predominantly in Western and Northern Europe, have reached a certain saturation point regarding FDI, and their growth has slowed. This is very similar to the findings of another study [52]. Of course, the reasons may also lie in protecting their assets, where countries have sought to de-globalize specific processes [1, 53]. On the other hand, the decline in efficiency was not high even in these countries. This suggests a high stability of FDI flows in the EU as a political grouping. The situation is different in countries that joined the EU later, and their attractiveness to foreign investors (especially in the industrial sector) remains high. The main reasons include relatively low labor costs, the quality of the workforce, geographically advantageous location, and political and economic stability, which is, to some extent, guaranteed by the EU. On the other hand, the nature and sectors into which FDI flows play a significant role. A large amount of FDI inflows into the EU is directed towards the tourism sector, as Western EU countries and Mediterranean countries are global leaders in tourism [54, 55].

It has been proven that FDI can contribute to developing economies in the context of the negative aspects of the COVID-19 pandemic [18]. For this reason, it is crucial to emphasize that the results suggested by this study are more than satisfactory for the EU, as there has not been a significant decline in FDI efficiency. This is why the EU is better prepared for further economic development.

## Conclusion

As anticipated, the impact of the COVID-19 pandemic, which occurred at the turning point of the years within our observed period, significantly affected the global economies, especially the inflow and outflow of international investments. Despite unfavorable conditions, some countries registered an increase in FDI efficiency during this period. Based on observations of the results of the DEA analysis, it can be evaluated that less economically developed EU countries (not least developed) tend to gain from the effects of FDI in times of crisis.

In general, we must state that the efficiency of FDI in EU countries was not significantly reduced in the early stages of the pandemic. It is evident that other effects also slow down with the slowing down of economies. This is precisely why we used the DEAmethod, which can take these criteria into account through mathematical modeling. In this study, we used FDI as the output variable, which is an unconventional approach, but clarifying the effects of FDI is desirable. We also included inward and outward FDI in the outputs, providing a comprehensive view of the development. For the future, more detailed research would be appropriate to consider using these variables in models separately. This is also the limitation of this research, which was intended to provide a comprehensive view of the effects of FDI in times of crisis in the EU.

Of course, this research can be conducted on other objects of study, and the results may indicate global and regional differences. However, the conclusion of our study is as follows: 1) there was no significant reduction in the efficiency of FDI in the EU in the first year of the pandemic, indicating that the EU appears to be an economically resilient region; 2) EU countries with lower economic levels even achieved an increase in FDI efficiency values, despite many restrictive measures and uncertainties in the market; 3) these positive changes in efficiency were primarily caused by positive changes in managerial efficiency, leading to the conclusion that decisions made by relevant authorities had positive effects on FDI flows in economically less developed EU countries.

These findings and considerations have been fully demonstrated in decomposing productivity changes into managerial and technological efficiency. Most countries that joined the EU in 2004 later dominate in managerial efficiency, which may be due to their industrial orientation, where this efficiency is crucial. This aspect also implies a lower focus on research and development than more developed EU countries. Historical economic contexts cannot be overlooked either, where Eastern EU countries were heavily oriented towards planned economies until the 1990s. Although technologies were at a relatively good level, managerial governance lagged or was limited to the tasks of centralized plans.

The contributions of this study are evident both theoretically and practically. Theoretically, it summarizes the current findings of other authors concerning already confirmed aspects of FDI processes in the relevant literature. This contribution is most significant in terms of the current examination of the impacts of the COVID-19 pandemic on changes in FDI processes. Currently, data is available that helps us understand these effects. A thorough examination and synthesis of these findings is the most important theoretical contribution of this study.

The use of DEA models in examining and benchmarking DEA is not common practice. Our results also indicated that this model has the potential to capture the actual situation in the FDI market robustly. Practically, the contribution of this study is mainly in the areas of crisis management and its position in EU policies. It appears that countries do not respond to significant changes caused by crises in the same way. Therefore, in the future, individual EU bodies, such as the European Commission, must take timely but targeted measures that will be communicated with the relevant governments of individual countries (including ministries and other executive government bodies) to protect the attractiveness of the environment for both the inflow and outflow of FDI. Additionally, we have found further differences in that some countries are more sensitive to changes in processes that can be influenced by managerial (governmental) decision-making–predominantly Eastern European countries. Conversely, some countries are more sensitive to shifts in the overall technological curve, and these are also more stable in the area of FDI. The findings presented in the previous sections are essential for complementing a holistic view of crisis management in the field of FDI management and other sectors of economic processes.

## Supporting information

S1 Table Input and output variables. (DOCX)
