# Supplementary material for: Empirical research of foreign direct investments efficiency in the European Union on the edge of pandemic outbreak
Source: PLoS One. 2025 Jan 8;20(1):e0313161. doi: 10.1371/journal.pone.0313161 (PMC11709250; doi:10.1371/journal.pone.0313161)
Supplement: S1 Table — (DOCX) [file pone.0313161.s001.docx]

| Year | Country | GFCF | Energy_cons_ind | Energy_cons_serv | Human_cap | FDI Inward Stock | FDI Outward Stock | GDP |
| --- | --- | --- | --- | --- | --- | --- | --- | --- |
| 2019 | Austria | 99.22 | 7542.72 | 2647.70 | 4148.00 | 196.73 | 234.99 | 397.52 |
| 2019 | Belgium | 115.49 | 10334.86 | 4607.34 | 4708.00 | 577.14 | 676.43 | 478.16 |
| 2019 | Bulgaria | 11.46 | 2678.81 | 1267.91 | 3121.00 | 52.72 | 3.04 | 61.56 |
| 2019 | Croatia | 11.95 | 1172.43 | 835.41 | 1631.00 | 29.42 | 0.99 | 55.57 |
| 2019 | Czech Republic | 61.07 | 6613.44 | 3191.90 | 5126.00 | 171.33 | 45.13 | 225.57 |
| 2019 | Denmark | 66.14 | 2257.33 | 1917.08 | 2637.00 | 121.48 | 217.68 | 310.48 |
| 2019 | Estonia | 7.05 | 459.27 | 467.03 | 619.00 | 27.94 | 10.01 | 27.73 |
| 2019 | Finland | 57.12 | 10963.15 | 3015.00 | 2411.00 | 85.82 | 146.47 | 239.85 |
| 2019 | France | 572.29 | 27500.35 | 21910.34 | 26281.00 | 868.69 | 1532.82 | 2437.64 |
| 2019 | Germany | 742.22 | 55649.35 | 27533.76 | 39955.00 | 934.74 | 1775.77 | 3473.35 |
| 2019 | Greece | 19.38 | 2587.61 | 2135.47 | 3811.00 | 45.15 | 19.24 | 183.25 |
| 2019 | Hungary | 39.57 | 4460.46 | 2050.44 | 4406.00 | 93.26 | 31.48 | 146.11 |
| 2019 | Ireland | 191.07 | 2248.46 | 1818.49 | 2177.00 | 1152.37 | 1085.93 | 356.53 |
| 2019 | Italy | 323.20 | 24928.49 | 18192.30 | 22579.00 | 444.51 | 557.03 | 1796.63 |
| 2019 | Latvia | 7.10 | 852.76 | 568.82 | 863.00 | 17.89 | 2.18 | 30.65 |
| 2019 | Lithuania | 10.48 | 1113.76 | 626.71 | 1314.00 | 20.85 | 4.80 | 48.86 |
| 2019 | Netherlands | 172.81 | 13043.89 | 6771.31 | 8077.00 | 1491.69 | 2089.69 | 813.06 |
| 2019 | Poland | 97.62 | 16492.20 | 7808.03 | 16012.00 | 234.93 | 25.42 | 533.60 |
| 2019 | Portugal | 38.82 | 4618.96 | 2359.34 | 4571.00 | 165.41 | 60.93 | 214.37 |
| 2019 | Romania | 50.45 | 6661.06 | 1962.14 | 8320.00 | 99.20 | 2.42 | 223.16 |
| 2019 | Slovak Republic | 20.30 | 3463.68 | 1222.22 | 2535.00 | 60.95 | 4.73 | 94.05 |
| 2019 | Slovenia | 9.50 | 1322.21 | 451.99 | 961.00 | 17.98 | 7.46 | 48.40 |
| 2019 | Spain | 249.89 | 20642.73 | 10310.56 | 19417.00 | 763.22 | 626.27 | 1244.38 |
| 2019 | Sweden | 116.40 | 10891.75 | 4024.80 | 4791.00 | 340.85 | 410.49 | 476.87 |
| 2020 | Austria | 95.77 | 7322.45 | 2569.84 | 4090.00 | 194.06 | 234.08 | 379.32 |
| 2020 | Belgium | 109.32 | 10023.24 | 4469.98 | 4687.00 | 635.93 | 677.66 | 456.89 |
| 2020 | Bulgaria | 11.75 | 2646.35 | 1079.93 | 3015.00 | 59.72 | 3.43 | 61.33 |
| 2020 | Croatia | 11.20 | 1171.21 | 757.38 | 1618.00 | 32.07 | 1.25 | 50.19 |
| 2020 | Czech Republic | 56.34 | 6554.35 | 2987.05 | 5066.00 | 188.77 | 56.21 | 215.25 |
| 2020 | Denmark | 69.90 | 2304.25 | 1822.35 | 2618.00 | 135.13 | 244.65 | 312.52 |
| 2020 | Estonia | 8.23 | 407.97 | 471.45 | 606.00 | 34.45 | 11.12 | 26.83 |
| 2020 | Finland | 57.55 | 10248.23 | 2809.78 | 2381.00 | 96.90 | 166.01 | 238.00 |
| 2020 | France | 528.85 | 25798.38 | 20340.88 | 26151.00 | 968.14 | 1721.80 | 2302.86 |
| 2020 | Germany | 735.87 | 54188.97 | 26772.16 | 39537.00 | 1059.33 | 1977.24 | 3367.56 |
| 2020 | Greece | 19.27 | 2523.50 | 1903.49 | 3766.00 | 51.80 | 21.86 | 165.33 |
| 2020 | Hungary | 36.60 | 4431.35 | 1999.98 | 4352.00 | 100.99 | 36.87 | 136.62 |
| 2020 | Ireland | 147.94 | 2166.93 | 1829.12 | 2150.00 | 1350.05 | 1206.73 | 372.87 |
| 2020 | Italy | 295.66 | 23861.10 | 16557.57 | 22129.00 | 485.84 | 596.16 | 1656.96 |
| 2020 | Latvia | 7.22 | 870.92 | 550.36 | 848.00 | 20.46 | 2.52 | 29.43 |
| 2020 | Lithuania | 10.42 | 1024.34 | 576.21 | 1290.00 | 23.71 | 5.10 | 49.51 |
| 2020 | Netherlands | 170.43 | 13118.37 | 6466.40 | 8101.00 | 2890.58 | 3797.60 | 800.10 |
| 2020 | Poland | 86.94 | 15921.17 | 7580.29 | 15978.00 | 248.73 | 26.60 | 523.67 |
| 2020 | Portugal | 38.18 | 4503.03 | 2159.50 | 4529.00 | 183.56 | 63.38 | 200.09 |
| 2020 | Romania | 52.18 | 6437.03 | 1834.29 | 8196.00 | 107.53 | 2.72 | 218.86 |
| 2020 | Slovak Republic | 18.07 | 3138.18 | 1108.48 | 2483.00 | 63.99 | 5.34 | 92.08 |
| 2020 | Slovenia | 8.86 | 1260.00 | 415.35 | 959.00 | 20.42 | 8.67 | 46.92 |
| 2020 | Spain | 227.60 | 18840.56 | 9451.50 | 18851.00 | 853.29 | 624.84 | 1121.95 |
| 2020 | Sweden | 117.81 | 11562.25 | 4007.56 | 4740.00 | 408.82 | 464.54 | 475.67 |
